# A comparative evaluation of medicine package inserts for oral antidiabetic agents in Palestine

**DOI:** 10.1186/s12889-019-7379-8

**Published:** 2019-08-02

**Authors:** Malak Eshtayeh, Asia Draghmeh, Sa’ed H. Zyoud

**Affiliations:** 1 Department of Medicine, College of Medicine and Health Sciences, An-Najah National University, Nablus, 44839 Palestine; 2 Poison Control and Drug Information Center (PCDIC), College of Medicine and Health Sciences, An-Najah National University, Nablus, 44839 Palestine; 3 Department of Clinical and Community Pharmacy, College of Medicine and Health Sciences, An-Najah National University, Nablus, 44839 Palestine; 4 Clinical Research Centre, An-Najah National University Hospital, Nablus, 44839 Palestine

**Keywords:** Patient package insert, Anti-diabetic agents, Palestine

## Abstract

**Background:**

Patient package inserts (PPIs) should provide accurate, sufficient, and clear information for patients as well as health care professionals. The goal of this study was to evaluate and compare the PPIs of local and imported anti-diabetic agents in the Palestinian market.

**Methods:**

Eighteen leaflets were collected and analysed based on the completeness of 31 criteria using a scoring method, then the quantity of information was assessed by applying word counting of 17 headings and subheadings. Statistical comparisons of the word count for all products were performed using the Mann-Whitney U test with mean ranks. Then the mean ranks for differences in word counts were adjusted to calculate the fold-difference statistic by dividing the higher mean rank by the lower mean rank.

**Results:**

In general, the PPIs of imported agents scored better than local PPIs, but none of the inserts fulfilled the whole criteria. Thirteen out of thirty-one criteria were available in all products. None of these agents had provided any information about duration of use, instructions to convert tablets into liquids forms, pharmacokinetics, or shelf life. Moreover, mechanism of action and maximum dose were deficient in all local PPIs (0.0%), while they were included in 37.5 and 62.5% of imported PPIs, respectively. Furthermore, 90.0% of local PPIs lacked information about drug dose, 80.0% didn’t mention any instructions regarding effects on ability to drive or possibility of tablet splitting, and 60.0% didn’t involve orders about possibility of tablet crushing. Local PPIs provided inadequate and less detailed instructions regarding many aspects, since the estimated mean rank of local and imported PPIs demonstrated a range of difference from 1.04-fold for missing dose to 2.64-fold for warning and precautions.

**Conclusions:**

Significant differences were being identified, with excellence being assigned to imported PPIs. So, it is worth suggesting some necessary modifications in PPI topography and sequence structure of local diabetic agents. Experts in Palestinian Ministry of Health should implement regulatory guidelines to improve the quality and quantity of information provided by local PPIs. This optimisation could become a step forward toward optimal health practice in our society.

## Background

It is generally agreed that patients need reliable and scientific information about their disease and prescribed medications. Patient package inserts (PPIs) are probably the most accessible source of drug information for both patients and health care professionals, as PPIs always accompany prescription only and over the counter (OTC) drugs, this is true especially in areas with limited healthcare resources [1].

The cohesion and the harmony of information available in PPIs affects patient compliance and faith in taking or rejecting the drug [2, 3]. Moreover, patients who read PPIs are found to have an increasing level of awareness and knowledge about drugs issue [4]. Despite all efforts, PPIs still reflect major drawbacks, as patients may feel frightened from the listed drug side effects in PPI and have confusion while reading medical jargon [5, 6].

This highlights the necessity for PPIs to be clear and comprehensible for the general public. Several studies regarding PPIs were conducted around the world, and found that the quality and quantity of information mentioned in PPIs greatly influences patient compliance and satisfaction [7]. The Palestinian Pharmacy Practice law stipulates that PPIs should come with all medications and to be available in Arabic and English languages as well [8]. However, the Palestinian law does not create strict guidelines that adjust the design and types of information included by PPIs [9].

Diabetes mellitus has emerged as a major health problem with serious medical complications and it’s considered one of the leading causes of mortality among the Palestinian population. Because of this, diabetic agents were chosen to represent the domain of the current study.

Globally, the number of patients who suffered from diabetes mellitus is highly increased over the last three decades, this number is projected to rise over 350 million by 2030, making it one of the most challenging public health issue to all nations, the causes of diabetes epidemic can be referred to changes in genetic background, aging, and sedentary lifestyle including (physical inactivity, irregular diets) which figures the higher level of obesity among populations worldwide. This growing medical burden achieved current prevalence of about 15% in Palestine. The chronicity characteristics of DM implicates a major rule of patients’ families, health care providers and pharmaceutical companies to support those patients to follow up their disease and encourage them to adhere to their lifelong medications, diabetic patients are graved to be fully educated about their illness and directed towards the proper use of their medications regarding exact daily dose and timing of their diets to help achieving tight glycemic control, minimising the morbidity and mortality rates associated with toxic overdose [10] and so promising maximum drug benefit. Despite the importance of doctors’ instructions to their patients about how to use their prescribed agents, many of those patients are highly suspected to forget such verbal instructions which underscores the need for reliable written reference to be checked out by patients when necessary, so clear PPIs represent a powerful background that can be followed by diabetic patients when doctors’ instruction are found to be inconsistent or being confused. Several pharmacological treatment options for DM from both local and imported companies are available in Palestinian pharmacies. Therefore, we performed this study to evaluate and compare the PPIs of diabetic drugs that manufactured locally versus the PPIs of their imported counterparts.

The main goal of the conducted study was to assess the availability of key information on PPIs of local diabetic agents in Palestine, and to suggest regulatory guidelines to be complied with by local pharmaceutical companies to ensure submission of optimal and high-quality PPIs. Despite the wide range of information sources regarding medications that are currently available for many patients, package inserts still reflect the most reliable and accessible source, not only for patients but also for pharmacists and health care providers, because this printed leaflet is the only source of scientific, essential, and approved information about a drug. This is important to aid in the success of the treatment plan and ensure safe and effective medical practice while using both prescriptions only and OTC medicines. Recently, educating patients about their prescribed drugs has gained increased attention and awareness by doctors as well as pharmacists, since many studies reported that noncompliance rates with therapies are consistently high due to the gap in communication between the physician and patient [11]. Therefore, several programs have been arranged to counsel patients about prescription agents, and one of these programs was the introduction of patient package inserts [5] which hoped to intensify and reinforce verbal instructions that were often vague and forgettable for many patients.

## Methods

### Study design

A cross-sectional comparative study was used to assess and compare the PPIs of diabetic drugs that manufactured locally versus the PPIs of their imported counterparts. The study was carried out in West Bank, Palestine, between January and March 2018.

### Selection of PPIs

Inclusion criteria of anti-diabetic drugs were as follows: (1) have in minimum one imported analog; (2) produced by at least one local pharmaceutical company; (3) registered in the pharmacy department of West Bank’s Palestinian Ministry of Health (MOH) (4) obtainable in oral solid form (capsules and tablets).

Exclusion criteria involved combination drugs, agents that are unlisted in the Department of Pharmacy of the Palestinian MOH, and injection forms of insulin, which were excluded due to unavailability of competitive local products, whereas the Palestinian markets are embedded only with imported forms. Dependent on those criteria, four active ingredients: metformin, glimepiride, sitagliptin, and glibenclamide, were picked. They were available as 18 leaflets: 10 of them were manufactured locally and the residual eight were equivalent agents produced outside the Palestinian authority areas. Table 1 displays the trade names of the local and imported antidiabetic agents that were included in the study.

**Table 1:** Trade names of the local and imported anti-diabetic medications that were included in the study

Anti-diabetic agent	Local products	Imported products
Metformin hydrochloride	Diamet®	Glucophage®
	Glucomet®	Metformin_Teva®
		Glucomin®
Glimepiride	Amiran®	Amaryl®
	Glimaryl®	Glimepiride- Teva®
	Amarrex®	
Glibenclamide	Glucocare®	Daonil®
	Declamide®	Gluben®
	Gluconil®	
Sitagliptin	Sitaglu®	Januvia®
	Juvesta®	

### Collection of PPIs

PPIs were collected from 25 community pharmacies that were primarily located in Nablus, Ramallah, Tubas, and Jenin. The pharmacies were selected conveniently (non-random), pharmacists offered some help by identifying the most popular diabetic medications available in the Palestinian markets.

### Evaluation of PPIs

Assessments of the collected package inserts were done using a set of criteria [9, 12–16]. A scoring method was used in order to evaluate the availability of information in our sample of PPIs by establishing a check list to test the availableness of 31 information statements that include: active ingredient, adverse drug reactions (ADR), brand name, contraindications, drug dose, drug-drug interactions, directions for use, drug food interactions, date of last revision, duration of usage, effect on ability to drive and use machines, geriatric considerations, inactive ingredients, indications, instructions to convert tablets or capsules into liquid forms, lactation considerations, maximum dose, mechanism of action, missing dose, name and address of manufactures/distributers, overdose and management, pediatric considerations, pregnancy considerations, pharmacokinetics information, possibility of crushing and mixing with food or beverages, possibility of tablet splitting, shelf life, sources of information, storage, therapeutic class, and warnings and precautions. The presence of any of the previously mentioned statements in either PPI was scored as one and its absence as zero, and then the calculation of total score for all anti-diabetic agents was done. Another method was used to evaluate PPIs by applying simple word counting of the major headings [11, 12, 17–19], which is an important method for evaluation of PPIs. Therefore, 17 heading and subheadings of both local and imported PPIs were evaluated for the quantity of information by using simple word counting. The remaining 14 criteria were neglected when the word counting criteria didn’t seem to be meaningful or due to deficiency in local or/and imported PPIs.

Data were assessed by two authors independently (ME, AD). The data was elicited and interpreted based on eligibility criteria. Any disagreements between the review authors were resolved through discussions with the principle investigator (SZ). No worthy differences arose between the reviewers.

### Statistical analysis

Analyses were done using the SPSS (Statistical Package for the Social Science; SPSS Inc., Chicago, IL) version 15. Continuous data were expressed as the median and interquartile range, and categorical data were expressed as frequencies and/or percentages. Statistical comparisons of the word count for local and imported antidiabetic drugs for all products were performed using the Mann-Whitney U test with mean ranks. Then the mean ranks for differences in word counts were adjusted to calculate the fold-difference statistic by dividing the higher mean rank by the lower mean rank for the 21 headings and subheadings available in both local and imported PPIs. *P* <  0.05 was considered statistically significant.

## Results

### Patient information leaflets

The total number of anti-diabetic agents that were available in the Palestinian markets was 18. Ten of them were produced by local companies and the remainder were imported (Table 1). The 10 local agents were manufactured by four local pharmaceutical companies, distributed as A (*n* = 3), B (*n* = 3), C (*n* = 2), and D (*n* = 2) as shown in (Table 2).

**Table 2:** Scores of the thirty-one statements written in the leaflets inserted in the local and imported diabetic drugs

		Local Companies						
No.	Criteria	A (*n* = 3)	B (*n* = 3)	C (*n* = 2)	D (*n* = 2)	Total scores of Local products *n* = 10 (%)	Total scores of Imported products *n* = 8 (%)	Total *n* = 18 (%)
1.	Brand name	3	3	2	2	10 (100.0)	8 (100.0)	18 (100.0)
2.	Active ingredient	3	3	2	2	10 (100.0)	8 (100.0)	18 (100.0)
3.	Inactive ingredients (excipients)	0	0	0	0	0 (0.0)	8 (100.0)	8 (44.4)
4.	Therapeutic class	2	3	1	2	8 (80.0)	8 (100.0)	16 (88.8)
5.	Mechanism of action	0	0	0	0	0 (0.0)	3 (37.5)	3 (16.6)
6.	Indications	2	3	2	2	9 (90.0)	8 (100.0)	17 (94.0)
7.	Drug dose	1	0	0	0	1 (10.0)	6 (75.0)	7 (38.8)
8.	Duration of use	0	0	0	0	0 (0.0)	0 (0.0)	0 (0.0)
9.	Missing dose	3	3	2	2	10 (100.0)	8 (100.0)	18 (100.0)
10.	Maximum dose	0	0	0	0	0 (0.0)	5 (62.5)	5 (27.7)
11.	Directions for use	3	3	2	2	10 (100.0)	8 (100.0)	18 (100.0)
12.	Overdose and management	3	3	2	2	10 (100.0)	8 (100.0)	18 (100.0)
13.	Warning and precautions	3	3	2	2	10 (100.0)	8 (100.0)	18 (100.0)
14.	Effect on ability to drive and use machines	0	0	0	2	2 (20.0)	7 (87.5)	9 (50.0)
15.	Contraindications	3	3	2	2	10 (100.0)	8 (100.0)	18 (100.0)
16.	Adverse drug reactions	3	3	2	2	10 (100.0)	8 (100.0)	18 (100.0)
17.	Drug-drug interactions	3	3	2	2	10 (100.0)	8 (100.0)	18 (100.0)
18.	Drug-food interactions	0	0	0	0	0 (0.0)	8 (100.0)	8 (44.4)
19.	Pregnancy considerations	3	3	2	2	10 (100.0)	8 (100.0)	18 (100.0)
20.	Lactation considerations	3	3	2	2	10 (100.0)	8 (100.0)	18 (100.0)
21.	Pediatric considerations	3	1	0	2	6 (60.0)	8 (100.0)	18 (100.0)
22.	Geriatric considerations	1	0	0	0	1 (10.0)	0 (0.0)	1 (5.6)
23.	Possibility of tablet splitting	0	1	0	1	2 (20.0)	6 (75.0)	8 (44.4)
24.	Possibility of crushing and mixing with food or beverages	0	1	0	1	4 (40.0)	6 (75.0)	10 (56.0)
25.	Instructions to convert tablets or capsules into liquid forms	0	0	0	0	0 (0.0)	0 (0.0)	0 (0.0)
26.	Pharmacokinetic information	0	0	0	0	0 (0.0)	0 (0.0)	0 (0.0)
27.	Shelf life	0	0	0	0	0 (0.0)	0 (0.0)	0 (0.0)
28.	Storage	3	3	2	2	10 (100.0)	8 (100.0)	18 (100.0)
29.	Name and address of manufacturers/distributors	3	3	2	2	10 (100.0)	8 (100.0)	18 (100.0)
30.	Date of last revision	0	0	0	0	0 (0.0)	8 (100.0)	8 (44.4)
31.	References	0	0	2	0	2 (20.0)	0 (0.0)	2 (11.1)

A: Beit Jala Pharmaceutical Company

B: Pharmacare Pharmaceutical Co.

C: Birzeit Pharmaceutical Co.

D: Jerusalem Pharmaceutical Co.

### Evaluation scores and compliance to assessed criteria

The analysis of local and imported package inserts was carried out based on 31 criteria. In general, the PPIs of imported agents achieved higher scores than local PPIs, but none of the inserts fulfilled the whole criteria. Thirteen out of thirty-one criteria were available in all products including active ingredient, ADR, brand name, contraindications, directions for use, drug-drug interactions, lactation considerations, missing dose, name and address of manufactures/distributers, overdose and management, pregnancy considerations, storage, and warning and precautions. Four out of 31 criteria were not implemented by any of the local or imported leaflets including duration of use, instructions to convert tablets into liquids forms, pharmacokinetics, or shelf life.

Inactive ingredients, drug-food interaction, and date of last revision were covered by all imported PPIs (100.0%), which represent 44.4% of all PPIs versus none (0.0%) of local PPIs. Similarly, mechanism of action and maximum dose were deficient in all local PPIs (0.0%), while they were included in 37.5 and 62.5% of imported PPIs, respectively. Furthermore, 90.0% of local PPIs lacked information about drug dose, 80.0% didn’t mention any instructions regarding effects on ability to drive or possibility of tablet splitting, and 60% didn’t involve orders about possibility of tablet crushing; whereas, 75.0–87.5% of imported PPIs provided some information concerning these points.

In addition, local and imported PPIs scored similar percentages regarding therapeutic class and indication for use, which were mentioned in 80 and 90% of local products, respectively, compared to 100% of imported PPIs. Geriatric considerations and source of information were noted in about 15% of local PPIs, while they were absent in all imported counterparts.

### Words count evaluation

After comparison of word counting within the selected 17 major headings and subheadings of both local and imported PPIs as viewed in Table 3, 12 out of the 18 headings had significant differences in *p* values (< 0.05), as follows: ADR (*p* = 0.003), contraindications (*p* <  0.001), drug dose (*p* = 0.034), drug-drug interactions (*p* = 0.001), effect on ability to drive (*p* = 0.012), indications (*p* = 0.021), overdose and management (*p* = 0.001), pregnancy considerations (*p* = 0.012), possibility of tablet splitting (*p* = 0.034), storage (*p* <  0.001), therapeutic class (*p* = 0.021), and warning and precautions (*p* <  0.001). Moreover, the estimated mean rank of local and imported PPIs demonstrated a range of difference from 1.10-fold for missing dose to 2.64-fold for warning and precautions.

**Table 3:** Statistical differences between word counts for local and imported anti-diabetic medications

Variable	Local Median [Q1-Q3]	Imported Median [Q1-Q3]	Mean rank for local products	Mean rank for imported products	*P* value^a^	Fold difference in mean rank
Therapeutic class	3.5 [1.5–4.5]	8.0 [3.8–8.8]	6.90	12.75	**0.021**	1.85
Indications	21.8 [12.8–27.8]	39.0 [26.3–48.8]	6.90	12.75	**0.021**	1.84
Drug dose	0.0 [0.0–13.5]	43.0 [6.5–109.3]	7.10	12.50	**0.034**	1.76
Missing dose	38.5 [29.8–56.3]	44.5 [19.8–88.5]	9.35	9.69	0.897	1.04
Directions for use	57.5 [25.8–77.8]	58.5 [53.5–103.0]	8.60	10.63	0.460	1.24
Overdose and management	36 [35.5–48.3]	104.0 [56.3–155.5]	6.10	13.75	**0.001**	2.25
Warning and precautions	36.5 [29.8–77.0]	349.0 [302.3–441.3]	5.50	14.50	**< 0.001**	2.64
Effect on ability to drive and use machines	0.0 [0.0–19.5]	63.5 [42.0–77.8]	6.70	13.00	**0.012**	1.94
Contraindications	44.0 [29.5–59.3]	128.0 [103.3–292.0]	5.85	14.06	**< 0.001**	2.40
Adverse drug reactions	60.0 [36.3–128.5]	367.5 [291.3–401.5]	6.30	13.50	**0.003**	2.14
Drug-drug interactions	56.0 [34.3–83.8]	293.0 [108.5–467.5]	6.10	13.75	**0.001**	2.25
Pregnancy considerations	14.5 [9.3–23.8]	29.5 [21.8–42.0]	6.70	13.00	**0.012**	1.94
Lactation considerations	10.0 [7.3–20.5]	16.0 [13.3–25.0]	7.75	11.69	0.122	1.51
Pediatric considerations	6.5[0.0–14.5]	10.0 [9.0–15.3]	8.20	11.13	0.274	1.36
Possibility of tablet splitting	0.0 [0.0–1.0]	4.0 [0.75–5.8]	7.10	12.50	**0.034**	1.76
Possibility of crushing and mixing with food or beverages	0.0 [0.0–5.0]	4.5 [1.0–5.0]	8.30	11.00	0.315	1.33
Storage	17.0 [16.5–23.3]	101.5 [96.5–103.0]	5.50	14.50	**< 0.001**	2.64

^a^The *p* value is bold where it is less than the significance level cut-off of 0.05

## Discussion

In this study, many differences were found to be significant in both the completeness and presentation of information included in the package inserts between the local and imported drugs. It was observed that local PPIs were missing many essential forms of information and also provided inadequate and less detailed instructions in many aspects. It was found that date of last revision was lost from all local PPIs and available in all imported products, this greatly influences patient adherence to PPI instructions as patient confidence in their medicine subsequently decline after finding that the written information in the PPI is obsolete and out-of-date. However, none of the local or imported PPIs supplied any information about the duration of usage for the diabetic agents. This could be due to the chronicity of diabetes mellitus and so the needs for medications are lifelong, but at the same time most of PPIs informed the patients to continue on their medications even if there is an improvement unless directed by a physician.

Regarding the quantities of information available in the PPIs, significant differences were found in word counting between local and imported agents. One of these important variations was documented in the criterion of adverse drug reactions, where imported PPIs notably involved much more detailed information than local PPIs. Most of the imported leaflets classified these side effects as being: very common, common, rare, and very rare, while some leaflets preferred the numerical way of description the possibility of occurrence of a side effect. However, patients may be frightened while reading the long list of adverse drug effects. As a result, many leaflets state: “don’t be alarmed when reading the list of side effects, you may not suffer from any of them”. So many researchers adopted that the quality of information mentioned in the section of adverse drug reactions is what matters rather than the quantity, and some of them advised that delivering risk information numerically is better than verbal descriptors, because it allows the patient to know the accurate estimation of the likelihood of each side effect [20, 21].

Another clear difference was elicited in the warning and precaution part, where the mean rank for word count was 5.50 in all local PPIs compared to 14.50 in the imported PPIs. Imported PPIs explained in detail how to prevent, recognise, and manage lactic acidosis and hypoglycemia, which are considered the most dreadful side effects of biguanides and sulfonylureas, respectively. While local PPIs didn’t point out these topics, similar findings were noted in the drug-drug interactions section. There were no significant differences in word counts in the criterion of missing dose as well as directions for use, and they were mentioned in all local and imported PPIs. Our results reflect those of Munsour et al. (2017) who also recommeded that pharmaceutical manufacturers and policymakers should consider the cultural and language necessities of patients when designing PPIs to be a part of continuous quality management in their care setting [19].

Inadequate information in PPIs has been the scope for many studies around the world. A Palestinian study carried out 2017 comparing the PPIs of local and imported anti-hypertensive drugs concluded similar findings regarding adverse drug reaction, date of last revision, and inactive ingredients [17]. Another study conducted in Southern India indicated that the information mentioned in the analysed inserts were not sufficient to ensure safe drug utilisation, and it recommended to review current PPIs based on scientific guidelines [16]. Furthermore, a study was applied in the United States (US) to evaluate and compare the quality and quantity of patient information inserts provided with dispensed agents in the US, the United Kingdom (UK), and Australia. It documented that leaflets from the US lacked adequate information concerning contraindications and precaution information, while Australian leaflets achieved the best score, followed by leaflets from the UK [22]. PPIs have to provide accurate medical information to ensure safe and effective use of drugs and minimise medication errors. Therefore, they need to be effectively designed and updated, taking into account the literacy level of the patients.

### Strengths and limitations

Patient package inserts are the most easily and freely accessible source of drug information. They enrich the essential knowledge of patients as well as pharmacists. The content and cohesion of information in drug leaflets guarantees and improves patient compliance and faith in taking medication. As diabetes mellitus has become a global epidemic [23–25], the distribution of anti-diabetic drugs has increased dramatically among pharmacies. Our study is the first study to evaluate, compare, and analyse the purport of local anti-diabetic leaflets and those of drugs that are produced outside the area of Palestinian authority (abroad).

In fact, the most remarkable limitation in this study is the deficiency of universally approved criteria that would implement regulatory measures to control the layout and contents of PPIs. The criteria selected for the current study was derived from the literature, but it actually had some positive and negative aspects. Another point of concern is that the number of words doesn’t necessarily reflect the quality of information, brief and simple instructions may sometimes be easier to follow by patients. In addition, the study included only one drug family, anti-diabetic agents, which had a limited number of local types dispensed within the Palestinian pharmacies. Furthermore, the obtainability of anti-diabetic drugs was confined in West Bank due to restriction to reach Gaza strip and East Jerusalem pharmacies.

## Conclusions

In conclusion, this study was applied to assess and compare PPIs of local and imported anti-diabetic agents in Palestine. Significant differences were being identified, with excellence being assigned to imported PPIs. So, it is worth suggesting some necessary modifications in PPI topography and sequence structure of local diabetic agents. Experts in the Palestinian MOH may need to update the information of PPIs and review the language of some leaflets to make them more clearly comprehensible by lay patients. This optimisation will contribute to reduce medication misuse and could become a step forward toward optimal health practice in our society.

## Data Availability

The raw data of this research can be made available upon request from the corresponding author.

## Abbreviations

ADR: Adverse drug reactions
FDA: Food and Drug Administration
MOH: Ministry of Health
OTC: Over the counter
PPI: Patient package insert
SPSS: Statistical Package for Social Sciences
UK: United Kingdom
US: United States
